# Influences of Mating Group Composition on the Behavioral Time-Budget of Male and Female Alpine Ibex (*Capra ibex*) during the Rut

**DOI:** 10.1371/journal.pone.0086004

**Published:** 2014-01-08

**Authors:** Federico Tettamanti, Vincent A. Viblanc

**Affiliations:** 1 Department of Science for Nature and Environmental Resources, University of Sassari, Sassari, Italy; 2 Centre d’Ecologie Fonctionnelle et Evolutive, Equipe Ecologie Comportementale, Unité Mixte de Recherche 5175, Centre National de la Recherche Scientifique, Université Montpellier 2, Montpellier, France; Université de Sherbrooke, Canada

## Abstract

During the rut, polygynous ungulates gather in mixed groups of individuals of different sex and age. Group social composition, which may vary on a daily basis, is likely to have strong influences on individual’s time-budget, with emerging properties at the group-level. To date, few studies have considered the influence of group composition on male and female behavioral time budget in mating groups. Focusing on a wild population of Alpine ibex, we investigated the influence of group composition (adult sex ratio, the proportion of dominant to subordinate males, and group size) on three behavioral axes obtained by Principal Components Analysis, describing male and female group time-budget. For both sexes, the first behavioral axis discerned a trade-off between grazing and standing/vigilance behavior. In females, group vigilance behavior increased with increasingly male-biased sex ratio, whereas in males, the effect of adult sex ratio on standing/vigilance behavior depended on the relative proportion of dominant males in the mating group. The second axis characterized courtship and male-male agonistic behavior in males, and moving and male-directed agonistic behavior in females. Mating group composition did not substantially influence this axis in males. However, moving and male-directed agonistic behavior increased at highly biased sex ratios (quadratic effect) in females. Finally, the third axis highlighted a trade-off between moving and lying behavior in males, and distinguished moving and female-female agonistic behavior from lying behavior in females. For males, those behaviors were influenced by a complex interaction between group size and adult sex ratio, whereas in females, moving and female-female agonistic behaviors increased in a quadratic fashion at highly biased sex ratios, and also increased with increasing group size. Our results reveal complex behavioral trade-offs depending on group composition in the Alpine ibex, and emphasize the importance of social factors in influencing behavioral time-budgets of wild ungulates during the rut.

## Introduction

Many vertebrate species, such as polygynous ungulates, exhibit strict sexual segregation during a large part of the year, gathering only for mating [1]–[3]. During the reproductive season however, the formation of mixed groups (hereafter mating groups) enables individuals of different sex, age, experience and behavior to co-occur [2]–[8]. Because individuals may only allocate a finite amount of time to their daily activities, behavioral trade-offs brought about by social factors, such as mating group composition, may have significant consequences on individual fitness [5], [7].

In mating groups, important social factors that affect male and female behavioral time budgets and fitness include group size, adult sex ratio, and group member experience or age [5], [7]–[11]. For instance, biases in the sex ratio of mature adults (the operational sex ratio [11], [12]) may affect competition for available mates, with strong consequences on individual and group behavior [7]–[8], [11]. Similarly, individuals of different age are likely to differ in their body size and social rank [13]–[17], which in males is often strongly associated with fighting abilities and reproductive success [7]–[8], [10], [13], [16]. Thus, the age structure of males within mating groups, and especially the relative proportion of males of high *vs*. low social rank, is likely to have strong consequences on overall group behavior. Ultimately, by affecting the behavior of males and females, social factors may shape group stability and affect individual fitness [8], [18]. Hence, there is a need to refine our understanding of the proximate effects social factors have on ungulate behavior in mating groups, and how variability in the social composition of mating groups influences individual behavior, with emergent properties at the group level [19].

As most ungulates, Alpine ibex (*Capra ibex*) show strong sexual segregation during a large part of the year, but form mixed sex/age groups during the rut [4], [20]–[21]. Over the course of the reproductive season, ibex exhibit a fission-fusion social system allowing individuals to freely join or leave any given mating group [22]. During the rut, male ibex significantly reduce the amount of time spent foraging and lying to the benefit of mating-related activities, whereas females typically decrease the amount of time spent lying to the benefit of moving, standing and social activities [23]. Although the rut is generally considered highly energy-demanding for male ungulates [23]–[24], male-male agonistic behavior is surprisingly low in the Alpine ibex, owing to a pre-rut establishment of dominance hierarchies and the use of alternative mating tactics in dominant and subordinate individuals [13], [15], [22], [25]. Thus, in addition to adult sex ratio (and mate availability), the proportion of old to young males that differ in dominance rank in mating groups [13] is likely to strongly influence individual behavior, with emergent properties on male and female group behavior. Further, trade-offs between different activities (*e.g*. maintenance *vs.* agonistic behavior) are also likely to vary depending on group member composition. For instance one might expect subordinate males to invest more time into courtship behavior when group sex ratio is biased towards females, and/or when the ratio of dominant to subordinate individuals in a group is low.

The aim of this study was to investigate how social factors affected the overall time-budgets of male and female group members in mating groups of Alpine ibex. Specifically, we considered the effects of group size, adult sex ratio and the proportion of old and dominant (≥9-yrs) to younger (<9-yrs) males in 45 different mating groups where male and female group time-budgets were recorded, *i.e.* the average time spent by males and females resting, feeding, travelling, standing, courting, and in intra- or inter-sexual agonistic behavior. By potentially modulating mating opportunities (available partners) and/or the amount of harassment experienced by females, we expected group size and the adult sex ratio of mating groups to be important determinants of male and female behavioral trade-offs. In addition, we further considered whether the relative proportion of old (≥9-yrs) to young (<9-yrs) males in mating groups affected both male and female group behavior. Male ibex indeed reach full body size around 8.5–10.5 years of age [26], [27], and fully-grown males are dominant [25] and monopolize most of reproduction by courting and defending receptive females against tentative competitors [13]. For dominant males, competition for access to available mates might increase with increasing male-bias in group sex ratio, to a limit where monopolizing females may be too costly due to a high number of competitors [7], [28]. In addition, previous studies in ungulates have shown that male age-structure in mating groups may have consequent effects on female body condition and receptivity [29]–[32].

## Methods

### Study Area and Population Monitoring

This study was conducted from early December to mid-January 2009–2010, during the mating season of Alpine ibex [13], [27], [33]. Groups of ibex were monitored (see below) in the Southeastern part of the Swiss National Park (Val Trupchun; 2060 ha; 1840–2220 m altitude), where an ibex population is followed since 1998. In the Swiss National Park, habitat is dominated by a forest of Swiss stone pine (*Pinus cembra*) and larch (*Larix decidua*) below the tree line (2200 m altitude), and by alpine grasslands and bare rocks above. Annual precipitations amount to *ca.* 700–1200 mm. Both sides of the valley are characterized by avalanche runs and corridors shaped by fallen rocks that offer foraging grounds for ungulates out of high-forest areas [34]. In 2009–2010, the Val Trupchun population counted 72 males, 76 females and 4 juveniles born during the year for a total of 152 individuals (see [35] for more details about census). Although surprisingly low, the juvenile to female ratio observed in this valley is consistent with those observed in previous years, and potentially explained by the fact that juveniles experience high mortality rates due to diseases, adverse weather conditions and predation by golden eagles (*Aquila chrysaëtos*) [36], [47].

### Behavioral Observations

From the 10th of December 2009 to the 15th of January 2010, we performed behavioral observations on 65 different groups of Alpine ibex. Behavioral observations were performed between 9∶00 AM and 16∶30 PM. This amounted to a total of 97 h50 min of observations that were performed with spotting scopes (20–75×), using the scan sampling method described by [37]. The behavior of each individual present in a group was sampled every 3 min, during 1 h30 min-long sessions. During each scan, all individuals were distinguishable by their location in the group and specific characteristics (either ear tag, horn morphology or coat coloration), and were thus counted only once. Observations were distributed over all daylight hours and performed by two observers that selected 1 to 4 different groups a day. To insure that no group was observed twice in the same day, observations were performed concurrently in different areas (>1.5 km apart) by the two observers, and each observer selected groups separated by at least 1 km while walking in opposite directions. Groups were observed from a distance of 500–900 m. An ibex group consisted of an assemblage of individuals, each within 50 m of their nearest neighbor and in visual contact. Individuals’ sex was determined by horn morphology [27], [38]. Approximately 20% of the ibex population in the Swiss National Park is tagged (ear tags and/or colored collars) and monitored, and thus a small fraction of the individuals in our study were of known age. For unmarked males, age was estimated by counting the conspicuous annuli on the outer side of horns as previously described by [38]. For unmarked females, age was not estimated because we could not precisely distinguish annuli on their shorter horns from a long distance. Each individual present in the focal group was assigned to one of five age classes including females (unknown age), juveniles (born in June during the year of observation), and males. We assigned males to 3 different age classes: yearlings (1 yr-old males), young subordinate males from 2 to 8-yrs old, and fully-grown dominant males ≥9 yrs-old [13], [27]. Behaviors were defined according to [13]. We recorded 6 different behaviors for males and females. Common behaviors included (1) foraging, (2) standing vigilant (scanning the environment) or ruminating, (3) moving (walking and/or running), and (4) lying down flat on the ground either resting or ruminating. Besides, we also recorded (5) male courtship behavior (for a detailed description of those behaviors, see [13]) and (6) male and female agonistic behavior. Agonistic behaviors included male and female intra-sexual agonistic behavior (*i.e.* displacing another individual by slowly approaching it, locking or clashing horns), and inter-sexual (male-directed) agonistic behavior. In male-directed agonistic behavior, a female displaced a courting male located at her side away from her by horn contact or simply by initiating a head movement towards him (the male then backed-off before horn contact). Because our interest was to investigate the effects of group composition on the behavioral time budget of mating groups, we discarded from further analyses single-sex groups that were constituted of only female or male ibex. In total, our analyses were thus conducted on 45 mating groups, for all of which courtship behavior was recorded. Only a small fraction (13%) of the animals in this study were tagged and as a result, individuals were counted several times over the season. Thus, to avoid pseudo-replication, we chose to work at the group level averaging time budgets over mating groups only. Indeed, no group was sampled twice within a given day, nor ibex twice within a given scan. Our data indicate that the different mating groups sampled never had the same composition of individuals and were thus, in effect, different statistical units.

### Behavioral Time Budgets in Reproductive Groups of Alpine Ibex

Within each mating group, we established sex-specific behavioral time budgets by dividing the numbers of scans recorded per sex/behavior by the total number of scans recorded per sex. We used Principal Components Analyses (PCA) to characterize major behavioral axes of Alpine ibex male and female groups, as previously done in large ungulates [39], [40]. This method produces independent (orthogonal) behavioral axes (principal components, PCs) accounting for the non-independence of behavioral time budgets [39], [40]. We only retained principal components with eigenvalues >1 which together explained over 70% of cumulative percent variance [41]. This allowed reducing the dimensionality of our 7 behavioral categories to 3 major axes (PC1, PC2 and PC3; see results) both for males and females. PCAs were applied to correlation matrices of the proportion of time spent in the different behavioral categories, accounting for the fact that some behaviors were largely under-represented in the behavioral time-budget of Alpine ibex (for instance male-male agonistic behavior was extremely low as previously reported by [22]).

### Group Characteristics and Effects on Group Behavior

Alpine ibex group size was determined by summing all individuals recorded in each specific mating group. Because female age could not be determined precisely, all females were grouped in the same category. Adult Sex Ratio was then calculated as the relative proportion of potentially reproductive males to total female numbers in mating groups using the formula ASR = males_≥2-yrs_/(total female number+males_≥2-yrs_) [7], [11]. An ASR >0.5 indicates a male-bias, an ASR <0.5 indicates a female-bias, and an ASR = 0.5 indicates an even proportion of males and females in mating groups. In addition, we also calculated the proportion of old dominant (≥9-yr) *vs.* young subordinate (<9-yr) males in each mating groups as P_old_ = males_≥9-yr_/(males_2–8 yrs_+males_≥9-yr_). Summary statistics and Spearman rank correlations were used to describe the composition of mating groups during the reproductive season.

We investigated the effect of group social composition on group time-budget in male and female ibex separately. Male and female principal components (behavioral axes) were entered as dependent variables in separate Linear Models (LMs), and group size, adult sex ratio and P_old_ as independent explanatory variables. Quadratic influences of adult sex ratio and group size on male and female behavior were tested by including the quadratic terms adult sex ratio^2^ and group size^2^ into our models. Starting models included all two-way interactions between adult sex ratio, group size and P_old_ to account for the fact that the influence of those independent variables on Alpine ibex behavior might have been conditioned by one another. Minimal adequate models were obtained by stepwise model selection using the ‘stepAIC’ function (library ‘MASS’) in R. We retained models with the lowest Bayesian Information Criterion (BIC) as best fitting models. To help interpret significant interactions (see [42]), we report regression lines for each significant effect (*e.g.* group size) for the minimum, mean and maximum value of its interacting term (*e.g.* adult sex ratio). All statistical analyses were performed using the R.2.15.1 statistical software [43]. Mean estimates are given±SE, unless otherwise specified.

### Ethics Statement

The Research Manager of the Swiss National Park approved the protocol used in this study. No further approval by an Ethics Committee was required, as behavioral observations at a distance were non-invasive and the Alpine ibex a species classified of least concern (IUCN). Tagging of individual ibex were performed independently of the present study (both prior and during the study) by Rangers of the Swiss National Park, and approved by the Swiss Federal Veterinary Office.

## Results

### Composition of Alpine Ibex Groups during the Mating Season

Mean group size (±SD) across our 45 mating groups was 11.6±5.3 individuals and ranged from 4 to 36 Alpine ibex. The adult sex ratio was 0.54±0.16 and ranged from 0.25 in female-biased mating groups to 0.86 in male-biased mating groups. The proportion of older dominant males (≥9-yrs) relative to total male numbers in the group, ranged from 0.0 to 0.75, with a mean of 0.19±0.16. Group size positively correlated with total female number (*ρ* = 0.71, *P*<0.001) and total male number (*ρ* = 0.66, *P*<0.001) within the group, but was only weakly related to total juvenile number (*ρ* = 0.29, *P* = 0.06). As could be expected, total juvenile number positively correlated with total female number (*ρ* = 0.30, *P* = 0.04), but not total male number (*ρ* = −0.11, *P* = 0.47) within the group. Finally, group size did not correlate with adult sex ratio (*ρ* = −0.17, *P* = 0.25), or with the proportion of older dominant males in the group (*ρ* = 0.07, *P* = 0.63). Similarly adult sex ratio did not correlate with the proportion of older dominant males in the group (*ρ* = 0.03, *P* = 0.85).

### Behavioral Time Budgets in Reproductive Groups of Alpine Ibex

Differences in the behavioral group time-budget of males and females within the 45 observed mating groups are reported in Table 1. Both male and female groups spent a substantial amount of time grazing or standing vigilant, substantially less time lying or moving, and only very little time in intra-sexual aggressive behavior. On average, females spent more time grazing than males, whereas males spent more time standing vigilant, moving, and in intra-sexual agonistic behavior (Table 1). There was no significant difference in the amount of time spent by males or females lying.

**Table 1:** Mean (± SE) behavioral time budgets (%) of female and male Alpine ibex groups during the mating season.

	Female groups	Male groups	
Standing	27.1±3.5^a^	39.8±3.5^b^	
Lying	8.0±2.0^a^	5.5±1.7^a^	
Grazing	52.7±4.4^a^	30.2±3.4^b^	
Moving	10.1±1.1^a^	13.1±1.1^b^	
Courting	NA	10.6±1.5	
Intra-sexual agonistic behavior	0.2±0.09^a^	0.9±0.4^b^	
Inter-sexual agonistic behavior	1.8±0.8	NA	

Time budgets are calculated within the 45 different mating groups and averaged (see Methods). Columns not sharing the same superscript are different for *P*<0.05 (Wilcoxon tests).

### Principal Components Analyses of Behavioral Time Budgets

For both males and females, the first 3 axes of the PCA accounted for over 70% of the observed variance in behavioral time-budgets (Table 2). In males, the first axis (PC1) loaded strongly and inversely on grazing and standing, distinguishing them from other behaviors. The second axis (PC2) especially distinguished courtship behavior and male-male agonistic behavior, while the final axis (PC3) loaded strongly and inversely on lying and moving behavior. In female groups, PC1 was similar to males, and especially distinguished grazing, standing and lying from other behaviors. In contrast, PC2 loaded similarly on moving and female male-directed agonistic behavior, whereas PC3 inversely distinguished female-female agonistic behavior, moving and lying behavior from other behaviors (see Table 2).

**Table 2:** Independent orthogonal axes (PC1, PC2 and PC3) obtained from a principal component analysis describing the group behavioral time budget of male and female Alpine ibex (*Capra ibex*) during the mating season.

Males				Females			
	PC 1	PC 2	PC 3		PC 1	PC 2	PC 3
**Grazing**	**0.633**	−0.352	−0.352	**Grazing**	**0.676**	0.028	−0.148
**Moving**	0.287	0.199	**−0.532**	**Moving**	−0.131	**0.656**	**0.452**
**Courtship**	0.170	**0.670**	0.157	**Agonistic (F-F)**	0.171	0.052	**0.625**
**Agonistic (M-M)**	0.010	**0.617**	−0.008	**Agonistic (FM)**	−0.226	**0.651**	−0.306
**Lying**	−0.223	0.014	**0.746**	**Lying**	**−0.405**	−0.012	**−0.412**
**Standing**	**−0.662**	−0.073	−0.358	**Standing**	**−0.530**	−0.378	0.346
**SD**	1.37	1.16	1.10	**SD**	1.46	1.11	1.00
**% variance**	31.1	22.5	20.3	**% variance**	35.5	20.4	16.8

The standard deviation (SD) and the proportion of variance explained by each dominant axis are given.

**Figure 1:**
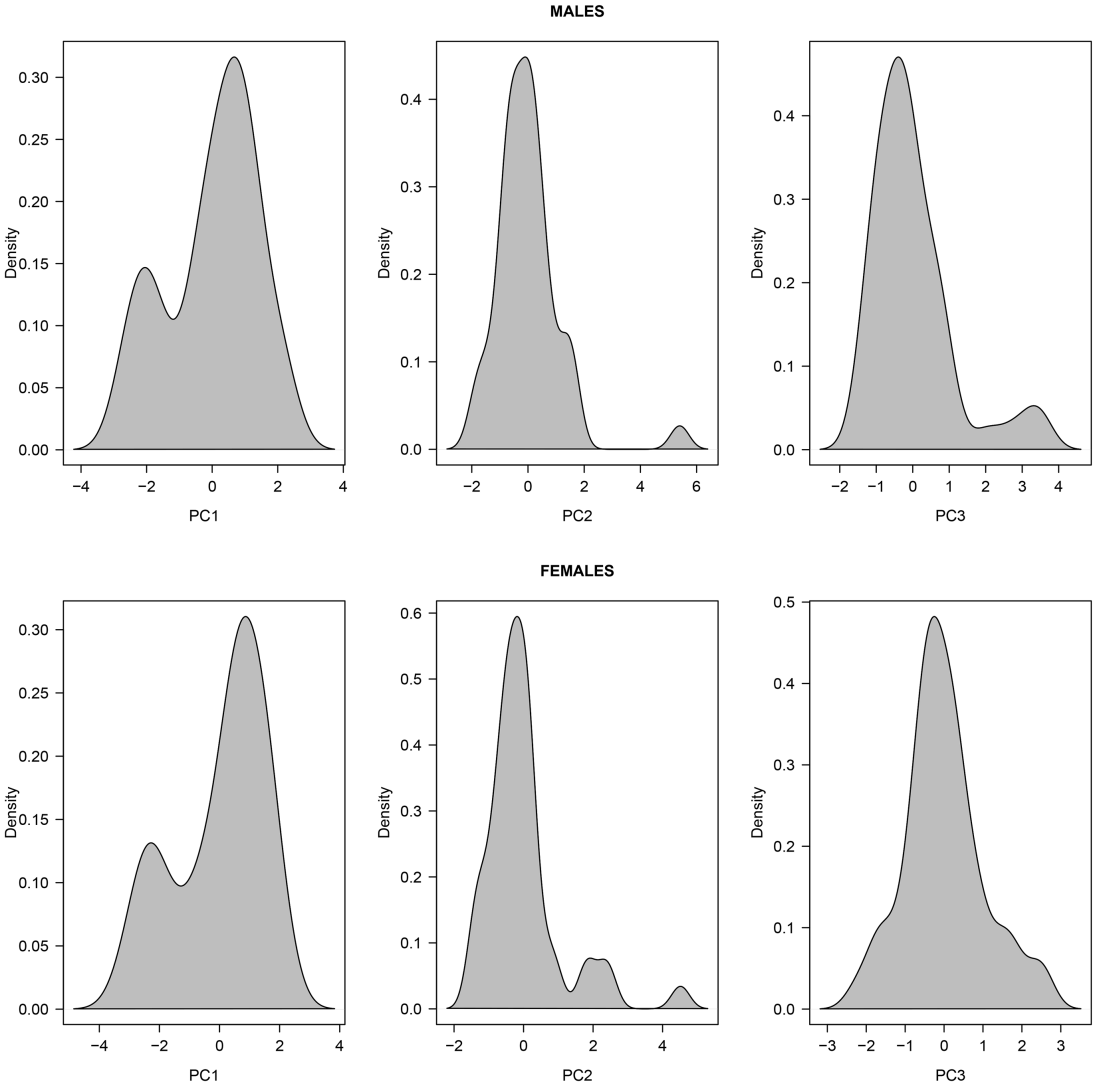
Kernel density distribution of the first three principal components (PC1, PC2, PC3) describing male and female group behavioral time budget in N = 45 different Alpine ibex mating groups. The loadings of the different principal components are given Table 2. Of note, PC1 was bi-normally distributed for both sexes. PC2 and PC3 were normally distributed, apart from a few extreme observations.

For both sexes the density distribution of principal components differed between PC1, PC2 and PC3 (see Fig. 1). Mostly, PC2 and PC3 were normally distributed, apart from a right skew in the distribution of PC2 (males and females) and PC3 (males) due to a few extreme observations. For instance, in one group, males spent 31.7% and 15.3% of their behavioral time budget in courtship and male-male agonistic behavior, respectively, explaining the high PC2 value (5.40) observed (Fig. 1). Thus, we ran subsequent model selection for PC2 and PC3 both with and without potential outliers to compare the outputs (see below). In contrast, PC1 was clearly bi-modal for both sexes. Optimal mixture modeling revealed multi-normal distributions for PC1 with means (±SD) of −2.09±0.39 and 0.64±0.80 for males, and means (±SD) of −2.38±0.32 and 0.67±0.80 for females. For both males and females, the first mode of PC1 distribution (low mean PC1) was especially due to groups that spent more time standing vigilant and less time grazing, than groups in the second mode (high mean PC1) (Fig. 1). Interestingly, 8 of the 11 mating groups (72.7%) with a low mean male PC1 (groups in the first mode) also had a low mean female PC1. Conversely, 32 of the 34 mating groups (94.1%) with a high mean male PC1 also had a high mean female PC1. Thus, for subsequent analyses of PC1, data sets were separated according to those different modes. We also modeled the probability for a mating group to end-up in either mode of male and female PC1 distribution, knowing its group size, adult sex ratio and proportion of older dominant males.

### Group Characteristics and Effects on Group Behavior

#### Male group behavior

**Figure 2:**
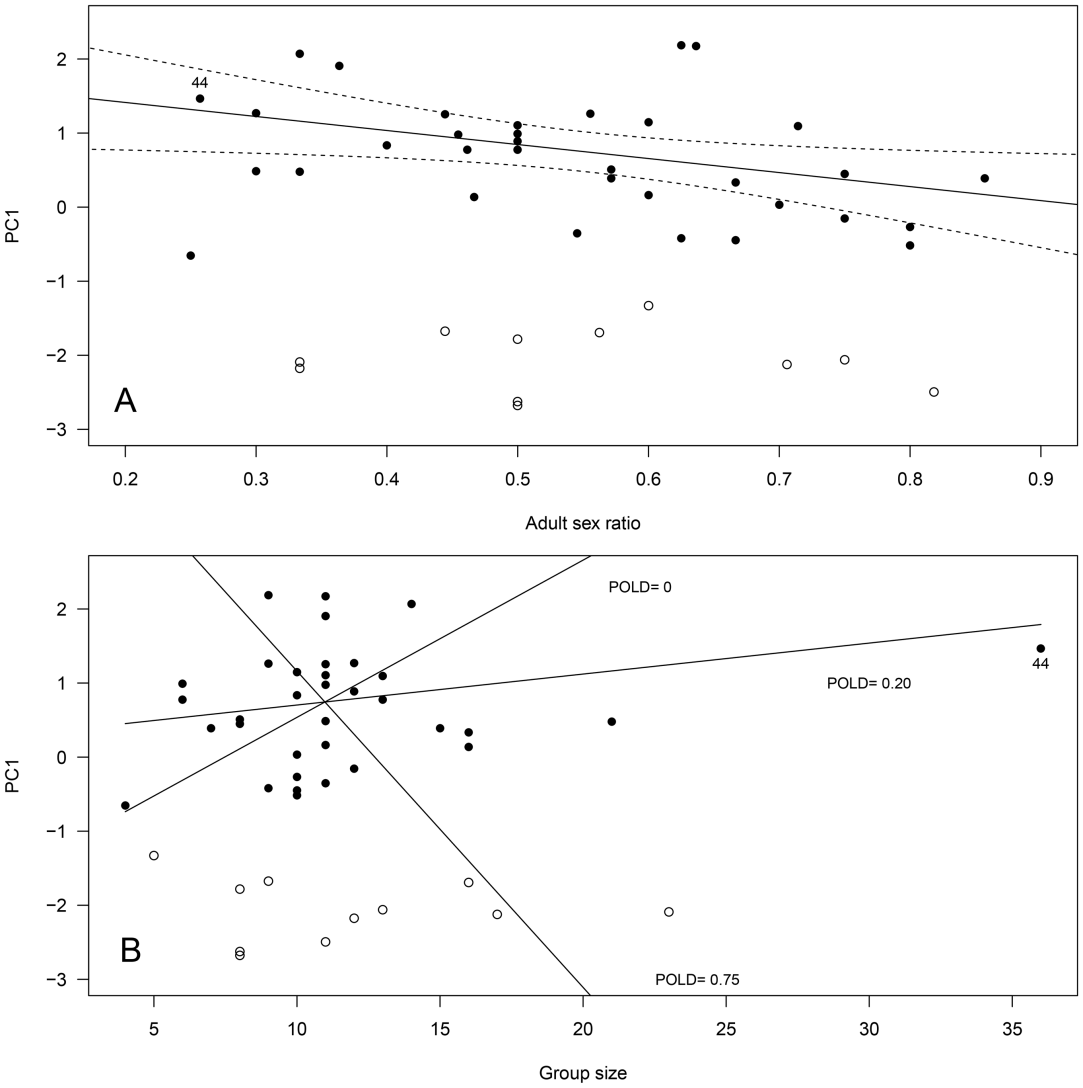
Influence of (A) adult sex ratio and (B) group size on male group behavior (Principal Component 1, PC1) in 45 different Alpine ibex mating groups. High values of PC1 predominantly reflected a high proportion of time spent grazing, and little time spent standing vigilant. The figure presents the best fitting model based on BIC for groups belonging to the second mode of bi-normal PC1 distribution data, excluding mating group n° 44 of especially high group size. Groups belonging to the first mode of PC1 distribution are figured as open circles and were not used in the analysis. Panel A: the regression line and 95% confidence intervals are given. Panel B: for illustrative purposes, the interaction between group size and the proportion of older males in mating groups (P_OLD_) is figured by showing the model regression lines only for the minimum, mean and maximum P_OLD_ observed in mating groups. However, statistics were run on the original continuous variable.

For males, PC1 was not affected by adult sex ratio, group size or the proportion of older males (≥9-yrs) in mating groups when considering the overall data. However, the analyses violated linear model assumptions due to the bimodal distribution of PC1. When restricting our analyses only to mating groups that belonged to the second mode of the bi-normal distribution (*N* = 34 groups with high mean male PC1; see Fig. 1), the best model retained adult sex ratio, the proportion of older males (≥9-yrs) in the group, group size, group size^2^, and a significant interaction between ‘group size x proportion of older males in the group’ (Table 3). The quadratic effect of group size was mostly due to one mating group (n° 44) of especially high group size (see Fig. 2B). Indeed, when this mating group was removed from the analysis, group size^2^ was not retained in the final model (Table 3). Thus, as adult sex ratio increased to favor males, males spent less time grazing and more time standing vigilant. Interestingly, male PC1 was also affected by group size, but the effect differed depending on the proportion of older males in the group. When this proportion was high (P_OLD_ = 0.75, Fig. 2), grazing behavior decreased (and standing vigilant increased) rapidly with increasing group size. In contrast, when this proportion was low (P_OLD_ = 0.20, Fig. 2), grazing behavior increased (and standing vigilant decreased) slightly as group size increased. When no older males were present (P_OLD_ = 0, Fig. 2), grazing behavior increased (and standing vigilant decreased) rapidly as group size increased. Unfortunately, we could not run the model selection on the first mode of male PC1 distribution (groups of low mean male PC1) because of low sample size (*N* = 11 groups). For those mating groups however, simple linear regressions of adult sex ratio, group size, group size^2^, or the proportion of older males in the group on PC1 were not significant (all *P*>0.73). Finally, adult sex ratio, group size and the proportion of older males in a mating group did not predict the likelihood of a given ibex group to be in one of the 2 modes of male PC1 distribution (GLM with binomial error; all *P*>0.57).

**Table 3:** Final model estimates explaining the variation observed along male behavioral principal components (PC1, PC2, PC3) in mating groups of Alpine ibex according to: group size, group size^2^, adult sex ratio (ASR and ASR^2^), and the proportion of old (≥9-yrs) males relative to all males of reproductive age (P_OLD_).

Response	Independent	Estimate ± SE	*t*	*P*
**PC1**	**Intercept**	−0.92±1.02	−0.89	0.38
*R^2^*_adj_ = 0.21, *F*_5,28_ = 2.74, *P* = 0.04, *N* = 35	**ASR**	−2.00±0.81	−2.47	0.02*
	**Group size**	0.30±0.12	2.51	0.02*
	**Group size^2^**	−0.004±0.002	−1.88	0.07
	**P_OLD_**	8.55±2.99	2.86	0.008**
	**Group size x P_OLD_**	−0.77±0.27	−2.87	0.008**
**PC1 (outlier removed)**	**Intercept**	−0.51±0.94	−0.60	0.55
*R^2^*_adj_ = 0.19, *F*_4,28_ = 2.89, *P* = 0.04, *N* = 34	**ASR**	−1.89±0.81	−2.34	0.03*
	**Group size**	0.21±0.08	2.55	0.02*
	**P_OLD_**	9.35±3.34	2.80	0.009**
	**Group size x P_OLD_**	−0.85±0.31	−2.77	0.009**
**PC 2**	**Intercept**	0.44±0.30	1.47	0.15
*R^2^*_adj_ = 0.07, *F*_1,42_ = 4.22, *P* = 0.046, *N* = 45	**Group size**	−0.05±0.02	−2.05	0.046*
**PC 3**	**Intercept**	−6.28±2.17	−2.89	0.006**
*R^2^*_adj_ = 0.22, *F*_4,40_ = 4.15, *P* = 0.007, *N* = 45	**ASR**	22.39±7.21	3.10	0.003**
	**ASR^2^**	−13.67±5.71	−2.39	0.02*
	**Group size**	0.22±0.08	2.82	0.007**
**PC 3 (outliers removed)**	**Intercept**	−1.84±0.92	−2.00	0.05
*R^2^*_adj_ = 0.11, *F*_3,36_ = 2.69, *P* = 0.06, *N* = 41	**ASR**	4.27±1.83	2.33	0.02*
	**Group size**	0.14±0.07	1.91	0.06
	**ASR x Group size**	−0.40±0.16	−2.51	0.02*

Starting models included all second order interactions. The presented models are those retained by stepwise model selection based on Bayesian Information Criterion (BIC). For PC1, only groups belonging to the second mode of the bi-normal distribution (of sufficiently large sample size) were included in the analyses. In addition, for PC1 and PC3, models excluding potential outliers are also presented (see text).

P<0.05,

P<0.01,

P<0.01.

**Figure 3:**
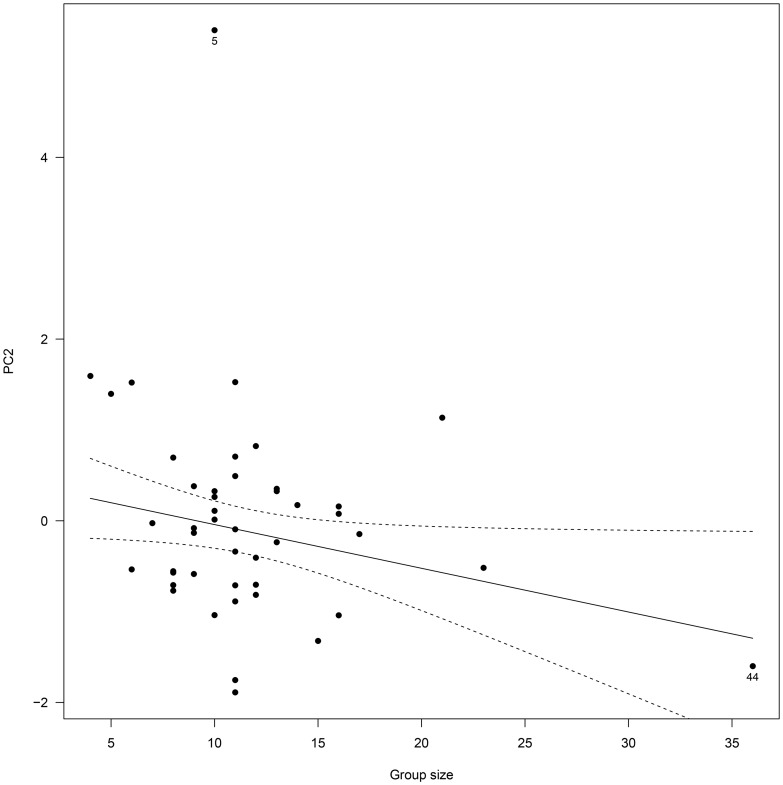
Influence of group size on male group behavior (Principal Component 2, PC2) in 45 different Alpine ibex mating groups. High values of PC2 predominantly reflected high male group time budget spent in courtship and intra-sexual (male-male) agonistic behavior. The figure presents the best fitting model (significant regression line and 95% confidence intervals) based on BIC for the data excluding mating group n° 5, of especially high PC2. Note that regression line is no longer significant when mating group n° 44 is also excluded from the analysis.

For male PC2, model selection did not retain any significant effect in the final model when considering the overall data. Removing a potential outlier of especially high PC2 from the analyses (mating group n° 5; Fig. 3), we found a significant negative effect of group size on the amount of time spent in courtship and male-male agonistic behavior (*t* = −2.05, *P* = 0.046) (Table 3; Fig. 3). However, the amount of variation explained was weak (R^2^ = 0.07) and the relationship was mostly due to one mating group of especially high group size (mating group n° 44; Fig. 3). Indeed, when this mating group was removed from the analysis, the effect of group size was no longer significant (*P* = 0.29).

**Figure 4:**
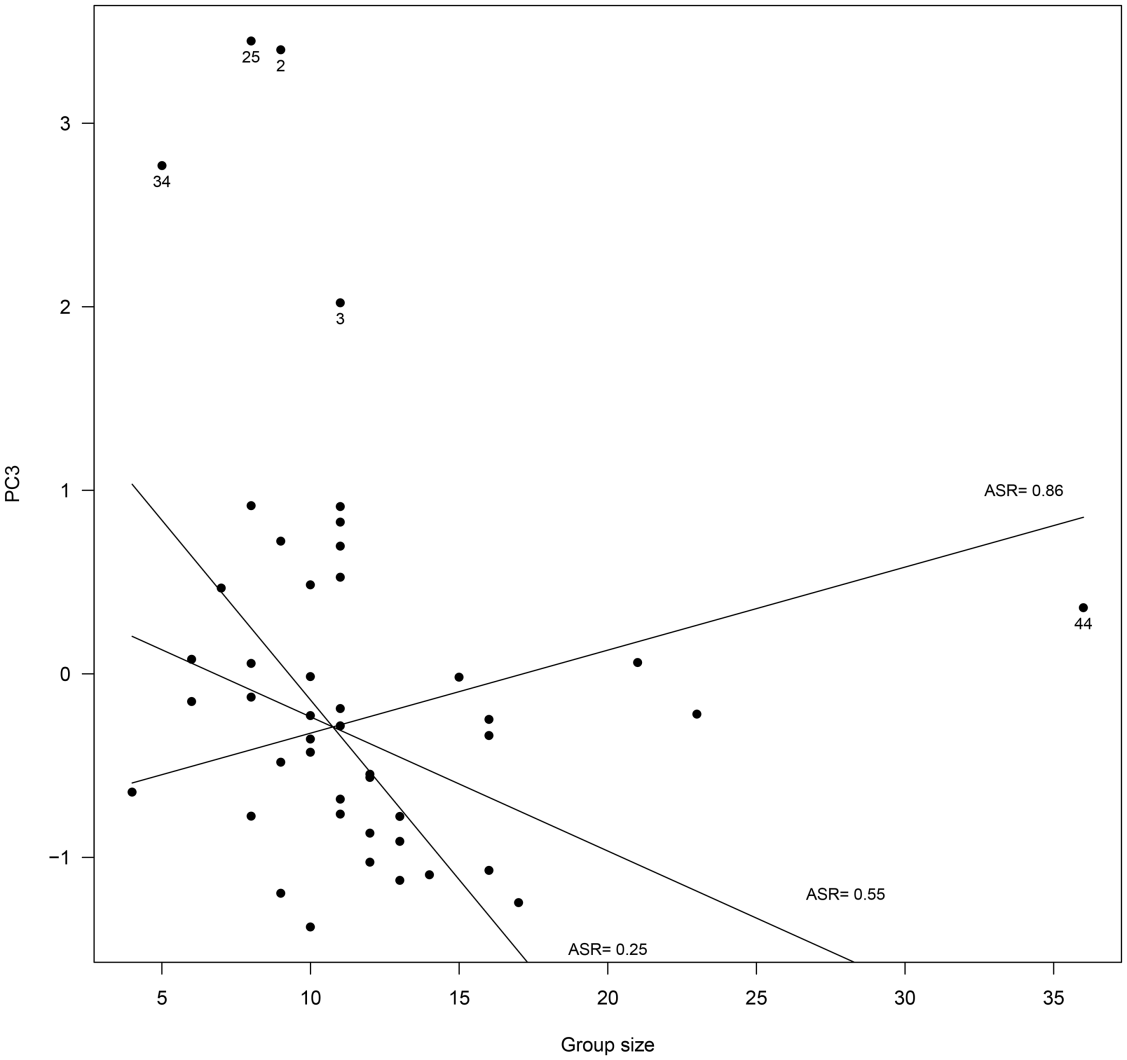
Influence of group size on male group behavior (Principal Component 3, PC3) in 45 different Alpine ibex mating groups. High values of PC3 predominantly reflected a high amount of time spent lying and little time spent moving. The figure presents the best fitting model retained by BIC for the data excluding mating groups n° 2, 3, 25, 34 and 44, of especially high PC3 (see text). For illustrative purposes, the interaction between group size and adult sex ratio (ASR) is figured by showing the model regression lines only for the minimum, mean and maximum (ASR) observed in mating groups. However, statistics were run on the original continuous variable.

In contrast, we found complex interacting linear and quadratic effects of adult sex ratio and group size on male PC3 (Table 3). The quadratic relationship however, was mainly due to mating groups of especially high PC3 (mating group n° 2, 3, 25 and 34; Fig. 3). Indeed, removing those groups from the analysis, the squared effect of adult sex ratio was no longer significant and the best fitting model only retained the linear interacting effects of adult sex ratio and group size on PC3 (Table 3). Thus, it appeared that group size affected male PC3 differently depending on adult sex ratio (Fig. 4). When adult sex ratio was biased to favor males (ASR = 0.86, Fig. 4), lying behavior increased and moving behavior decreased with increasing group size. In contrast, at equilibrated sex ratio, the inverse relationship was observed (ASR = 0.55; Fig. 4) and the effect was even more pronounced as sex ratio bias increased to favor females (ASR = 0.25; Fig. 4).

#### Female group behavior

**Figure 5:**
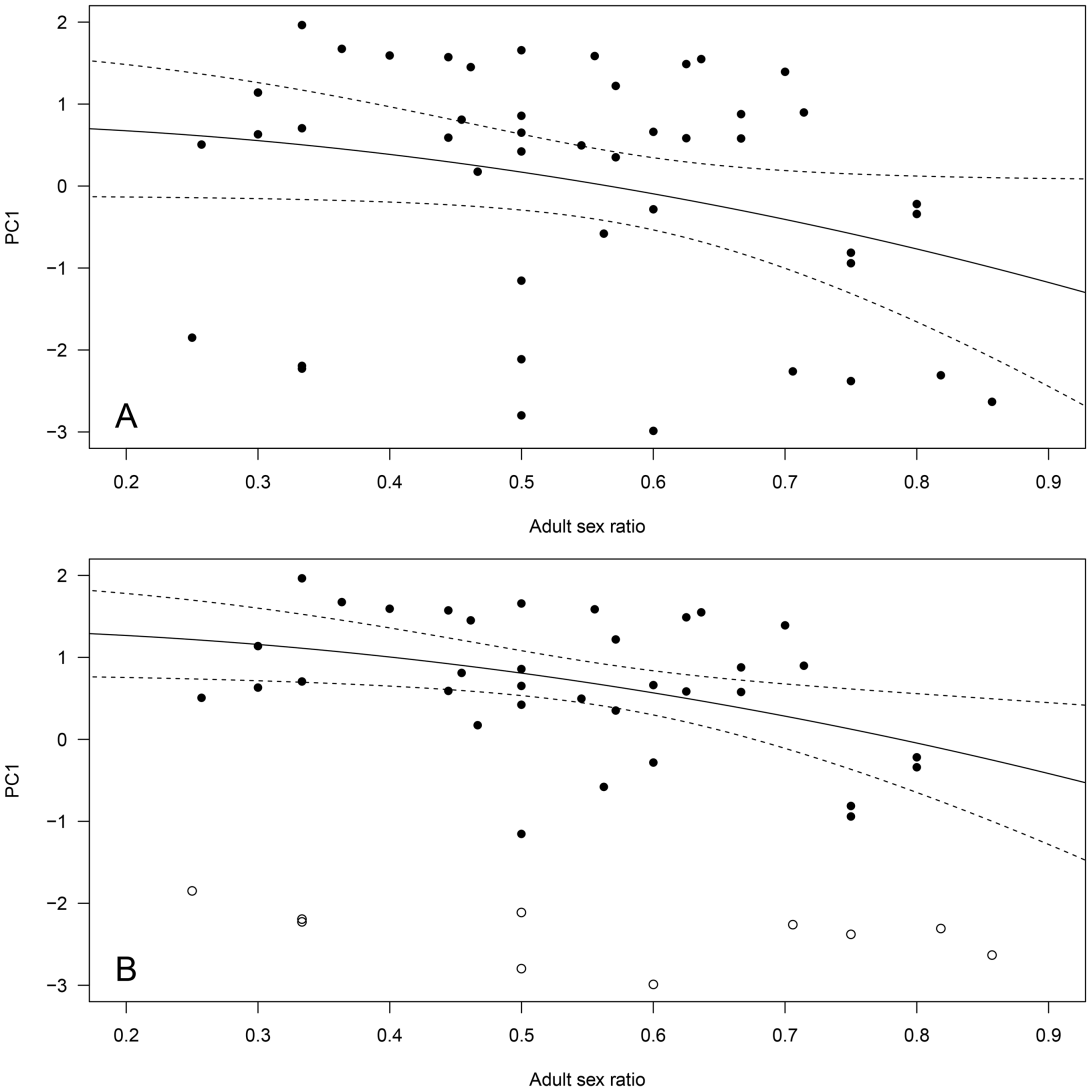
Influence of adult sex ratio on female group behavior (Principal Component 1, PC1) in 45 different Alpine ibex mating groups. High values of PC1 predominantly reflected high female group time budget spent grazing, and little time spent standing vigilant or lying. The figure presents the best fitting model (significant regression line and 95% confidence intervals) based on BIC for (**A**) the overall data (N = 45 mating groups); (**B**) the groups belonging to the second mode of bi-normal PC1 distribution (N = 35 mating groups, filled circles). Groups belonging to the first mode of PC1 distribution are figured as open circles and were not used in the analysis presented panel B.

For females, the best model fit on PC1 retained a negative quadratic effect of adult sex ratio when considering the overall data (−2.40 ASR^2^, *P* = 0.05). Again however, this model was not appropriate given the bimodal distribution of female PC1. However, restricting our analyses only to mating groups that belonged to the second larger mode of the distribution (*N* = 35 groups with high mean PC1; Fig. 1) yielded similar results (Table 4). Interestingly, for both models the effect of adult sex ratio on PC1 was similar in direction and magnitude. Thus, as adult sex ratio in mating groups increased to favor males, females spent less time grazing and more time standing vigilant (Fig. 5). Again, we could not run the analyses on the first mode of female PC1 distribution (groups of low mean female PC1) because of low sample size (*N* = 10). However, simple linear regressions of adult sex ratio or its quadratic term on PC1 were not significant (all *P*>0.19). Finally, adult sex ratio, group size and the proportion of older males in a mating group did not predict the likelihood of a given ibex group to be in one of the 2 modes of female PC1 distribution (GLM with binomial error; all *P*>0.57).

**Table 4:** Final model estimates explaining the variation observed along female behavioral principal components (PC1, PC2, PC3) in 45 mating groups of Alpine ibex according to: group size, group size^2^ and adult sex ratio (ASR and ASR^2^).

Response	Independent	Estimate ± SE	*t*	*P*
**PC1**	**Intercept**	1.35±0.28	4.83	<0.001***
*R^2^*_adj_ = 0.16, *F*_1,33_ = 7.28, *P* = 0.011, *N* = 35	**ASR^2^**	−2.19±0.81	−2.70	0.011**
**PC 2**	**Intercept**	6.17±1.75	3.53	0.001**
*R^2^*_adj_ = 0.26, *F*_3,41_ = 6.25, *P* = 0.001, *N* = 45	**ASR**	−21.90±6.04	−3.62	<0.001***
	**ASR^2^**	20.55±5.41	3.80	<0.001***
	**Group size**	−0.07±0.03	−2.50	0.02*
**PC 3**	**Intercept**	2.74±1.46	1.88	0.07
*R^2^*_adj_ = 0.38, *F*_4,40_ = 7.68, *P*<0.001, *N* = 45	**ASR**	−19.31±5.27	−3.66	<0.001***
	**ASR^2^**	16.19±4.69	3.45	0.001**
	**Group size**	0.34±0.08	4.35	<0.001***
	**Group size^2^**	−0.009±0.002	−4.08	<0.001***

Starting models also included the proportion of old (≥9-yrs) males (relative to all males of reproductive age) in mating groups, and all second order interactions. The presented models are those retained by stepwise model selection based on Bayesian Information Criterion (BIC). For PC1, only groups belonging to the second mode of the bi-normal distribution (of sufficiently large sample size) were included in the analyses. Statistics excluding potential outliers are reported in the text.

P<0.05,

P<0.01,

P<0.01.

**Figure 6:**
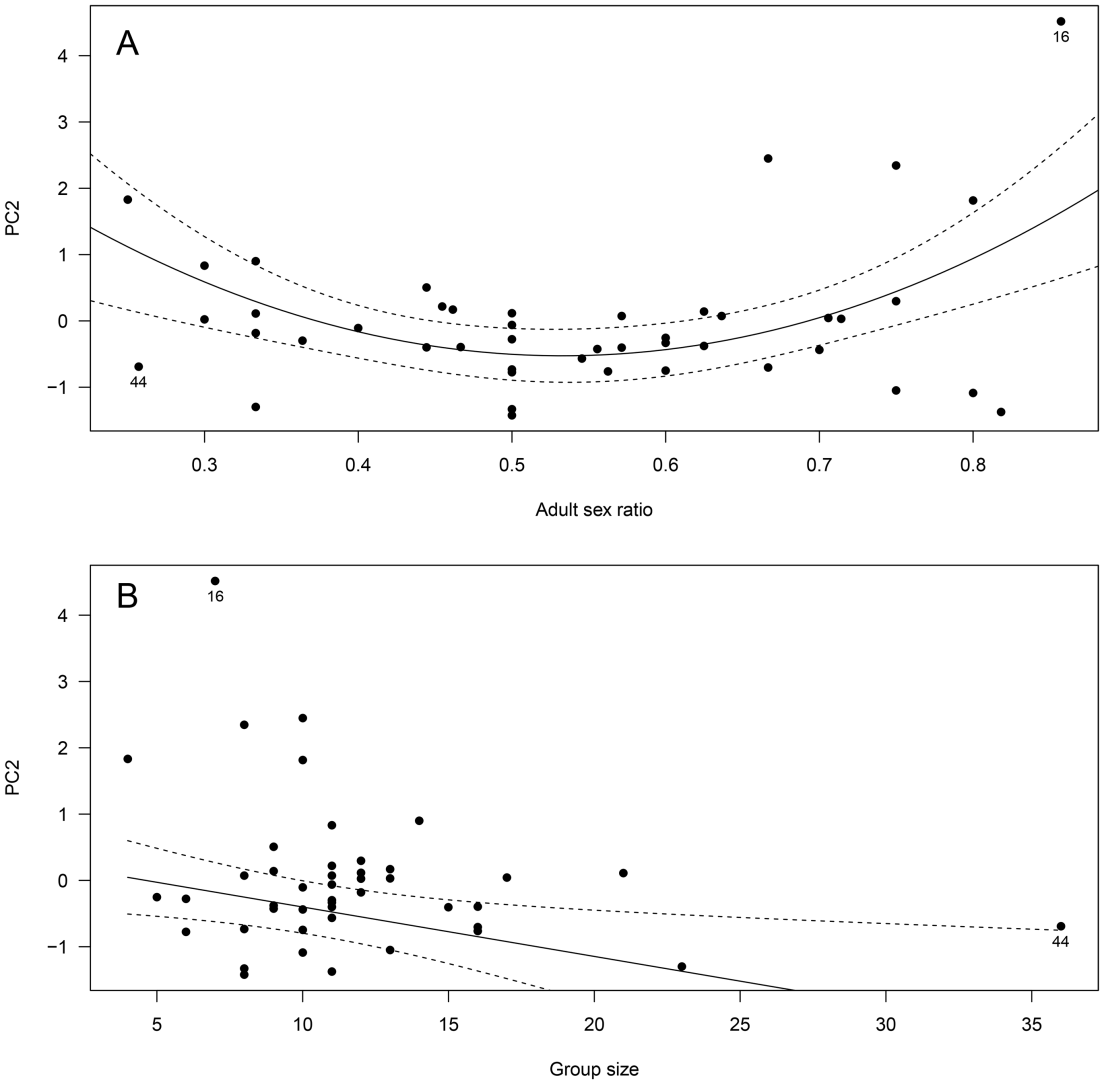
Influence of (A) adult sex ratio and (B) group size on female group behavior (Principal Component 2, PC2) in 45 different Alpine ibex mating groups. High values of PC2 predominantly reflected high female group time budget spent in moving behavior and in inter-sexual (male-directed) agonistic behavior. The figure presents the best fitting model (significant regression lines and 95% confidence intervals) based on BIC for the overall data (N = 45 mating groups). Potential outliers (mating groups n° 16 and 44) are shown as filled circles (see text for statistics without those mating groups).

For PC2, the best model fit retained significant linear and quadratic effects of adult sex ratio, and a significant negative effect of group size (see Table 4; Fig. 6A and 6B). Removing potential outliers (mating groups n° 16 and 44; see Fig. 6A and 6B) from the analysis did not change the results for adult sex ratio (−13.61 ASR, +12.21 ASR^2^, all *P*<0.05). Not surprisingly however, the effect of group size on PC2 was no longer significant (*P* = 0.12). Thus, when adult sex ratio was highly biased towards males or females, females increased the amount of time spent moving and the amount of time spent in male-directed agonistic behavior. In contrast, those behaviors decreased at equilibrated sex ratios (Fig. 6A). The amount of time spent moving and in male-directed agonistic behavior also decreased with increasing group size, but only when one especially large group (group n° 44; Fig. 6B) was kept in the analyses.

**Figure 7:**
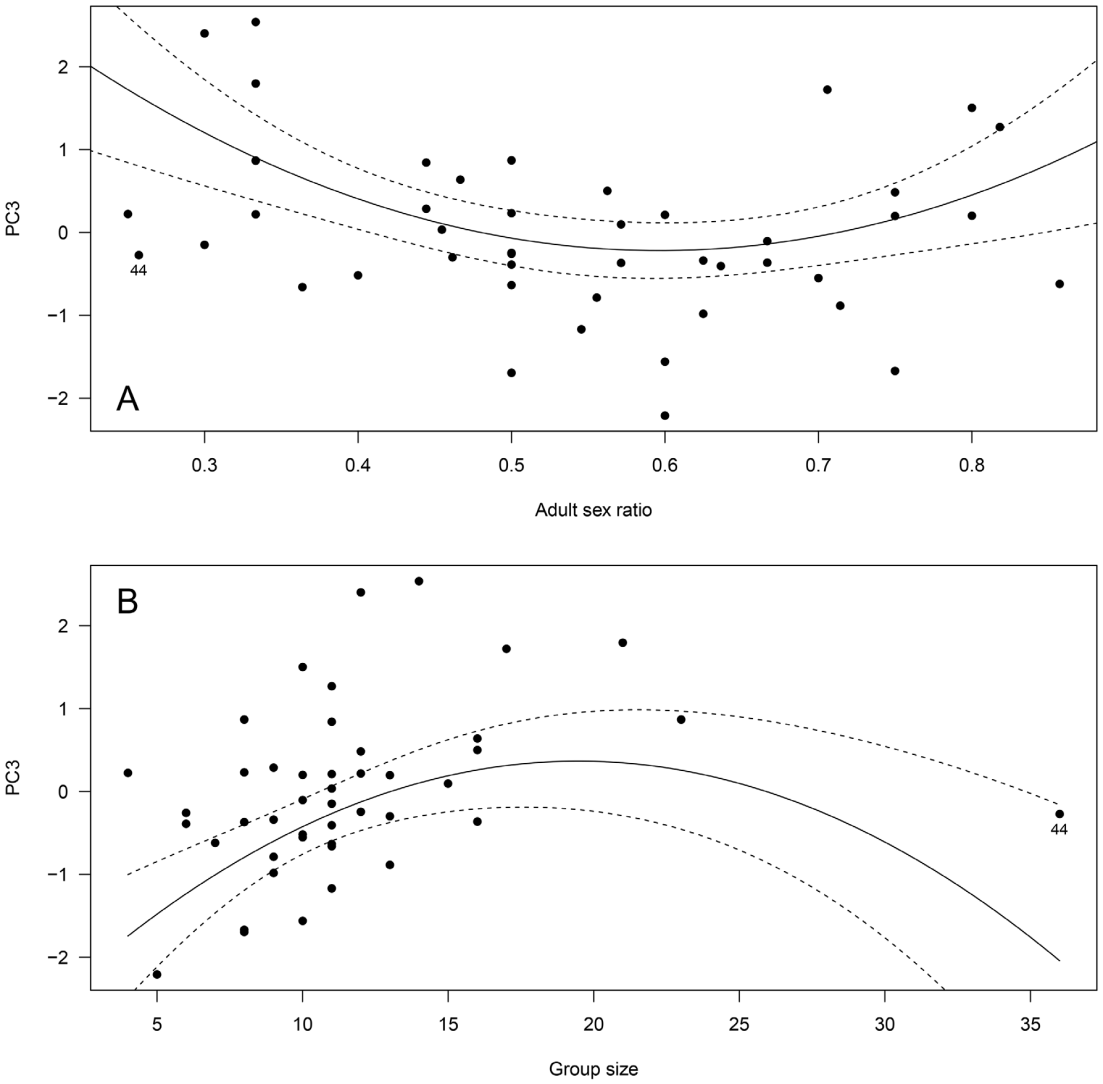
Influence of (A) adult sex ratio and (B) group size on female group behavior (Principal Component 3, PC3) in 45 different Alpine ibex mating groups. High values of PC3 predominantly reflected high female group time budget spent in intra-sexual (female-female) agonistic behavior and moving behavior, and little time spent in lying behavior. The figure presents the best fitting model (significant regression lines and 95% confidence intervals) based on BIC for the overall data (N = 45 mating groups). A potential outlier (mating group n° 44) is shown as a filled circle (see text for statistics without that mating group).

Finally, both adult sex ratio and group size had significant linear and quadratic effects on PC3 (Table 4, Fig. 7A and 7B). Again, excluding group n° 44 from the analyses (high group size) did not change the effects for adult sex ratio (−17.36 ASR, +14.74 ASR^2^, all *P*<0.01), but removed the quadratic effect of group size on PC3 retaining only a significant positive component (+0.12, *P*<0.001). Thus, as group size increased, females spent more time moving and in intra-sexual (female-female) agonistic behavior, and less time lying (Fig. 7B). Similarly, females spent more time in intra-sexual agonistic and moving behavior (and less time lying) at highly biased sex ratios, but showed the opposite pattern at more equilibrated sex ratios.

## Discussion

Our study underlines important effects of group social composition on the behavioral time-budget of Alpine ibex during the rut. Both male and female ibex group behavior indeed varied according to the underlying properties of mating groups. Importantly, because only a small fraction of individuals in our study were tagged and could be reliably re-identified over the season, we conducted our analyses at a group- (rather than individual-) level to avoid pseudoreplication. Nonetheless, it should be noted that the observed effects of social group composition on ibex behavior likely occurred first of all at an individual level. Further data on individually marked animals are thus required to determine how variable individual behavior in mating groups is, and what the precise links between individual-level behavior and emergent group properties are [19].

For both males and females, PC1 presented a bimodal distribution with some groups spending more time in vigilance than in grazing (low PC1), or the opposite (high PC1). Social factors (group size, adult sex ratio, and the relative proportion of older males within the group) did not predict the probability for a given group to be in either mode of PC1 distributions. However, it is interesting to note that the majority of the groups separated in male PC1 distribution were also those separated in female PC1 distribution. This suggests that male and female ibex in mating groups had synchronized activities in terms of grazing *vs.* vigilance behavior, consistent with the idea that behavioral synchrony may be important in maintaining group cohesion [44] even during the mating season. When focusing only on the second mode of PC1 distributions of larger sample size, we found that adult sex ratio, group size, and the proportion of older males (>9-yrs old) within a mating group had varying effects on the amount of group time-budget spent in vigilance/grazing by males and females. For both sexes, vigilance behavior increased at the detriment of grazing when adult sex ratio in mating groups was biased towards males. Previous studies in ungulates have found that vigilance/foraging trade-offs could be influenced by herd sex ratio, due to sex-specific differences in energy requirements [45] or variation in predation risk [46]. Predation of adult ibex however is negligible in the Alps [47], and it is unlikely that the effect of sex ratio on male and female vigilance/grazing behavior was a consequence of changes in individual predation risk. Rather, our results suggest that the time devoted to vigilance/grazing was highly responsive to the social environment.

Monitoring group social composition and/or the behavior of social conspecifics is an important component of ungulate social behavior [48], [49]. For females, vigilance group-behavior might have increased with adult sex ratio because of higher sexual harassment in male-biased groups [30], [50]. This suggestion is consistent with the fact that female group time-budget devoted to moving and agonistic behavior towards males (female PC2) also increased in male-biased mating groups. Although we recorded courtship behavior in all of the mating groups, we unfortunately have no data on female receptivity in this study. However, it is likely that in highly male-biased groups, female aggressiveness towards males reflected low female receptiveness. Alternately, sexually receptive females might have allocated less time to feeding and more time observing potential suitors when more males were present in the group [50], [51]. Indeed, recent studies have suggested that female mate choice may be more common in ungulates than previously thought (see [50] for a review). In this regard, it is interesting to consider whether female male-directed agonistic behavior in male-biased groups might have been an expression of female mate choice [50], used to displace non-favored suitors.

Although we observed very little female-female aggressiveness overall, we found that the group time-budget spent by females in intra-sexual agonistic behavior and moving behavior increased at the detriment of time spent lying (female PC3) with increasing group size and increasing sex ratio bias towards males or females (quadratic effect). Higher female-female agonistic behavior in larger groups may reflect feeding competition for limited high quality food patches [52] or better positions within the group. For instance, it would be interesting to know whether female aggressiveness allows to acquire more central positions within mating groups which might offer social thermoregulatory benefits [53]–[54] by sheltering individuals from detrimental weather conditions. The reason why female-female aggressiveness would increase at highly biased male or female sex ratios is unclear. Female aggressiveness and reproductive success are typically related to dominance status and age in mountain goats [55]–[57] and it is possible that in ibex, variation in the female composition of mating groups in terms of age/dominance affected female-female aggressiveness at highly biased sex ratios. However, we could not determine female age from a distance in the present study, and further investigations should thus consider whether similar patterns occur in mating groups where females of known age/dominance status are monitored.

For males, the increase in vigilance group time-budget (male PC1) that occurred in male-biased groups may have reflected a greater proportion of time allocated to monitoring conspecific behavior when numerous males were present in mating groups. Whereas the number of competitors within a group appeared to influence vigilance behavior, it is interesting to note that neither sex ratio nor group size (see below) had an effect on the amount of male group time-budget devoted to courtship or male-male agonistic behavior (male PC2). This result is consistent with the knowledge that strong social hierarchies are established prior to the mating season in Alpine ibex [25], dominant males monopolizing most of reproductive females. Male-male agonistic behavior is thus surprisingly low during the rut in this species [22], in agreement with our own observations. In addition, we found that male group time budget devoted to vigilance (but not to courtship or male-male agonistic behavior) also increased with group size at the detriment of grazing, but only when groups were constituted primarily of older (>9-yrs) males. In contrast, when mating groups were biased towards younger males, male group time-budget was actually more devoted to grazing than to vigilance behavior. Because dominance is related to body size in the Alpine ibex [25] and male ibex reach full body size at 8.5–10.5 years of age [26]–[27], it is likely that age-related dominance patterns in mating groups affected the amount of time devoted to monitoring the behavior of social competitors *vs.* time spent foraging (*i.e.* more time spent in vigilance when numerous dominant males were present in the group). Although we actually did find a negative effect of group size on the amount of time males devoted to courtship and male-male agonistic behavior (male PC2), this effect was essentially driven by a mating group of especially high group size (group n° 44) that spent little time in those behaviors and should thus be treated with caution. Indeed, mating groups of high group size are rarely observed during the rut (F.T. *personal observations*), and mating group n° 44 was observed at low altitude on Jan. 13^th^ following a particularly heavy snowfall event. Snow cover is known to strongly limit the movement capacity of Alpine ibex [33], [58] and may have constrained individuals to move towards less snow-covered areas at lower altitudes, gathering into a larger group. Under those conditions, it is possible that males may have been less motivated to engage into courtship or male-male aggressive behavior, as Alpine ibex males are known to decrease their investment in mating behavior in high snow cover conditions [15]. A similar explanation may also hold true for females, explaining why females in this mating group invested relatively little time moving or in male-directed agonistic behavior (low female PC2).

Finally, we found that male group time-budget spent lying increased at the detriment of time spent moving with increasing group size, but only when groups were especially biased towards males (male PC3). A high male-bias may have limited the opportunities for male-female social interactions [7], explaining why male movements generally decreased in such groups. Interestingly, this suggestion is somewhat supported by the observation that the opposite trend occurred in highly female-biased groups (males group time budget increased in time spent moving at the detriment of time spent lying).

Because the mating season of Alpine ibex occurs during winter at high altitude in the Alps, it is characterized by harsh environmental conditions. Indeed, both males and females spent a substantial portion of their time budget grazing, although females dedicated significantly more time to this activity than males, consistent with the idea that food and nutrient availability are essential determinants to female ungulate reproduction [58]–[64]. What is perhaps surprising is that relatively little time was spent by males in active courtship behavior, whereas proportionally more time was actually spent in vigilance or grazing behavior. Whereas this is likely due in part to the dominance hierarchies established before the rut, it should be kept in mind that physical activity linked to social interactions may have consequent effects on the energy budget of those animals. Thus, behavioral adjustments in male and female Alpine ibex may follow a trade-off minimizing costly activities while maximizing reproductive success depending on group social composition. Linking the energetics of social behaviors [65] to group composition and individual fitness during the mating season opens exciting perspectives for future research.
